# Advances in Optical Contrast Agents for Medical Imaging: Fluorescent Probes and Molecular Imaging

**DOI:** 10.3390/jimaging11030087

**Published:** 2025-03-18

**Authors:** Divya Tripathi, Mayurakshi Hardaniya, Suchita Pande, Dipak Maity

**Affiliations:** 1 School of Health Sciences, University of Petroleum and Energy Studies, Dehradun 248007, India; 2 Integrated Nanosystems Development Institute, Indiana University, Indianapolis, IN 46202, USA; 3 Department of Chemistry and Chemical Biology, Indiana University, Indianapolis, IN 46202, USA

**Keywords:** optical imaging, fluorescent probe, molecular imaging, therapeutics, medical imaging, contrast agents

## Abstract

Optical imaging is an excellent non-invasive method for viewing visceral organs. Most importantly, it is safer as compared to ionizing radiation-based methods like X-rays. By making use of the properties of photons, this technique generates high-resolution images of cells, molecules, organs, and tissues using visible, ultraviolet, and infrared light. Moreover, optical imaging enables real-time evaluation of soft tissue properties, metabolic alterations, and early disease markers in real time by utilizing a variety of techniques, including fluorescence and bioluminescence. Innovative biocompatible fluorescent probes that may provide disease-specific optical signals are being used to improve diagnostic capabilities in a variety of clinical applications. However, despite these promising advancements, several challenges remain unresolved. The primary obstacle includes the difficulty of developing efficient fluorescent probes, and the tissue autofluorescence, which complicates signal detection. Furthermore, the depth penetration restrictions of several imaging modalities limit their use in imaging of deeper tissues. Additionally, enhancing biocompatibility, boosting fluorescent probe signal-to-noise ratios, and utilizing cutting-edge imaging technologies like machine learning for better image processing should be the main goals of future research. Overcoming these challenges and establishing optical imaging as a fundamental component of modern medical diagnoses and therapeutic treatments would require cooperation between scientists, physicians, and regulatory bodies.

## 1. Introduction

Optical imaging, which uses light to effectively illuminate biological structures and processes, has become a key medical technology. Since it can generate detailed, real-time images of tissues, organs, and cells without subjecting patients to ionizing radiation, this non-invasive modality has many advantages over conventional imaging methods. Optical imaging has various advantages, more than the ionizing radiation-based imaging techniques, as optical imaging is a method that utilizes light to visualize biological structure and processes while ionizing radiation-based technique requires high harmful radiation that diagnoses the disease and any biological structure. Researchers and clinicians can obtain deeper insight into physiological changes and disease processes by using optical imaging that uses a variety of techniques like fluorescence, bioluminescence, and multispectral imaging to capture cellular dynamics [1]. Moreover, optical imaging is particularly effective for assessing different soft tissue attributes. Since soft tissues absorb and reflect light, it can detect metabolic abnormalities, which are often early indicators of aberrant organ and tissue function [2]. The term optical imaging refers to the variety of methods that visualize tissue optical properties based on light absorption, scattering, and fluorescence emission using UV to NIR light and numerous optical disease detection probes have been developed and assessed for their ability to generate disease-specific optical signals in the tissue. These include biocompatible probes made from cyanine dyes, tetrapyrroles, lanthanide chelates, and other substances. Additionally target-specific probes are being developed based on specific biomarkers, making them useful for diagnostic purposes and crucial for the future as these non-invasive techniques [3]. Furthermore, the development of sensitive charge-coupled device (CCD) technology, laser technology, as well as sophisticated mathematical modeling of photon propagation in tissue has led to the emergence of novel optical imaging technologies. In addition, there are currently fast-growing 3D quantitative fluorescence-mediated tomography and fluorescence reflectance imaging and other wide ranges of novel optical contrasting techniques that are intended to produce molecular contrast within a living creature and are rapidly emerging in tandem with these technological advancements. Moreover, a new range of instruments is being developed for in vivo molecular diagnostics, which are being developed by the combination of technological developments in light detection and improvements of optical contrast medium [4]. Techniques such as endoscopy, optical coherence tomography (OCT), photoacoustic imaging, diffuse optical tomography (DOT), Raman spectroscopy, and super-resolution are extensively used among the existing optical imaging techniques.

**Figure 1:**
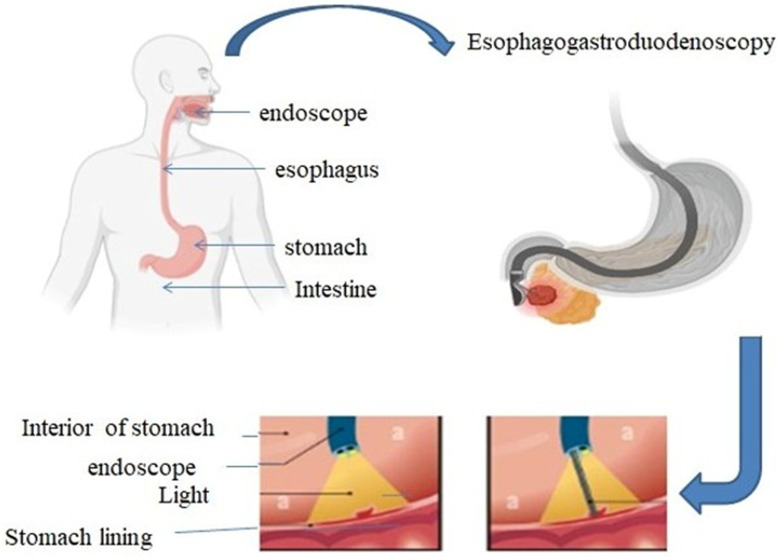
Gastroscopy is a procedure used to examine the esophagus, stomach, and upper portion of small intestine by inserting a flexible tube with a camera through the mouth. This process is employed to diagnose and treat various gastrointestinal disorders.

Endoscopy is a technique that is used to diagnose symptoms like pain, difficulty swallowing, or bleeding in the gastrointestinal tract. An endoscope is inserted through the patient’s mouth and into the digestive cavity (Figure 1), using a flexible tube equipped with a light source [3]. There are various other types of endoscopies based on the area of examination, like gastroscopy, which is used to investigate the esophagus, stomach, and part of the intestine. Colonoscopy examines the large intestine and rectum; bronchoscopy for the lungs and airways; cystoscopy to observe the bladder via the urethra; hysteroscopy to examine the uterus through the vagina; and capsule endoscopy which involves swallowing a capsule with a camera to capture images of the digestive tract. These endoscopic techniques can be used to diagnose various diseases like cancer, inflammatory bowel disease, and gastrointestinal tract-related diseases. Additionally, they can be used for tumor removal or for placing stents in narrowed passages to restore proper function [5,6,7].

Apart from the endoscopy, there are several other optical imaging techniques like optical coherence tomography (OCT), which enables images beneath the skin, making it particularly useful for detecting diseased tissue. OCT produces detailed images of subcutaneous tissue structures, aiding early diagnosis. Another method, diffuse optical tomography (DOT), is a non-invasive imaging approach that utilizes near-infrared light to assess blood oxygen saturation, total hemoglobin concentration, and other physiological parameters. DOT is widely used for applications such as breast cancer imaging, functional brain imaging, and stroke detection. Additionally, Raman spectroscopy has emerged as a powerful optical technique for identifying molecular compositions and detecting disease-related biochemical changes [3].

Optical imaging demonstrates high efficacy and specificity, particularly when contrast agents are employed. Contrast agents, also known as contrast media, are substances used in optical or medical imaging that enhance the visibility of internal body structures. Several types of contrast agents, such as iodine-based contrast agents, barium sulfate-based contrast agents, gadolinium-based contrast agents, and negative contrast agents, play a critical role in visibility during medical diagnosis. Their ability to enhance the contrast between various tissues makes it easier for medical personnel to spot anomalies and diagnose diseases more accurately [8,9]. To enable differential imaging of biological components, these agents usually contain fluorescent probes that absorb energy and emit light at a longer wavelength when exposed to light of a particular wavelength [10]. The capabilities of medical imaging have been significantly enhanced by the development of new optical contrast agents, particularly fluorescent probes, which are designed to improve sensitivity and specificity for detecting low-abundance biomarkers in various disorders, including cancer [11]. 

Optical imaging has many benefits, especially when it is combined with sophisticated contrast agents. This includes reducing radiation exposure to patients, enabling multiple imaging sessions, and capturing dynamic processes in real time to gain a better understanding of disease progression [12]. Optical imaging is useful for cancer screening as well as tumor location and resection by identifying human tumors. Optical imaging in neonatal care has shown potential in diagnosing conditions like stroke and intraventricular hemorrhage, and ongoing observation of very sick infants is being explored [13]. Because it can give us both qualitative and quantitative information at the cellular and molecular level, optical imaging is very useful for cancer identification. Three-dimensional imaging is becoming more common in this field, which improves tumor cell localization, distribution, and volume assessment. Optical imaging has many biomedical and clinical applications as it is a fast, sensitive, and non-invasive imaging modality [14,15]. Super-resolution microscopy and photoacoustic imaging are two merging optical technologies that have further advanced the field by enabling detailed molecular and cellular investigations at unprecedented depths and resolutions [16].

A key process in many optical imaging methods is fluorescence, which is the stimulation of fluorophore molecules followed by their emission of light as they settle back to their ground state [17]. This essential procedure not only enables the visualization of cellular components but also facilitates the development of complex fluorescent probes that can precisely target specific tissues or biomarkers [18]. Small fluorescent probes with high sensitivity and low biological interference have been the focus of recent research. With longer lifetimes and bigger Stokes shifts, these probes—like excimer-based fluorescent probes—are useful for many bioanalytical applications [19]. The development of probes that respond to changes in subcellular microenvironments or biomarkers further enhances their sensitivity and specificity for imaging within specific organelles, and fluorescent probes increase sensitivity and specificity by providing a strong detectable signal that distinguishes targets from surrounding tissues and increase specificity by incorporating targeting moieties that selectively bind to a specific biomarker or cellular structure [20,21].

In optical imaging techniques such as super-resolution microscopy, these probes have simplified the visualization of proteins at the nanoscale. In conclusion, the creation of fluorescent probes is advancing optical imaging and opening new avenues for the study of biological systems [22].

This review explores the recent developments in optical contrast agents, with a particular emphasis on fluorescent probes and how they relate to molecular imaging. This document is structured in the manner described below: First, an overview of optical imaging techniques will be provided before going into the details of the different kinds of contrast agents and fluorescent probes that are available. Subsequent sections will discuss the latest technological developments in the field, their clinical uses, and their significance in improving medical diagnoses and treatments. Together, these revelations will clarify how optical imaging technologies are transforming molecular biology and patient care, paving the way for novel diagnostic approaches that are tailored to personalized medicine.

## 2. Principle of Optical Imaging

Optical imaging primarily utilizes light to produce and project images of structures and objects in space. The application of optical imaging techniques enables the visualization of living cells or samples with enhanced contrast. The object under examination can absorb, scatter, or emit light, which helps in providing contrast and reveals specific properties of the subject under study [23].

**Figure 2:**
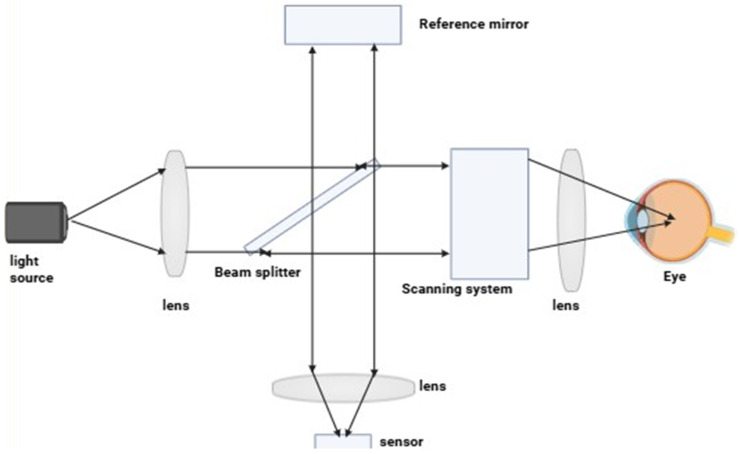
Illustration of a scanning optical imaging system. A light source produces a beam that is collimated by a lens and divided by a beam splitter into sample and reference arms. The reference arm sends a light to the stationary mirror, whereas the sample arm employs a scanning system and lens to focus light on the eye. Light reflected from each arm recombines at the beam splitter and is imaged onto a sensor through another lens, forming an interference pattern that is processed to produce a cross-sectional image of the internal structures of the eye.

The key principle of this optical imaging is the interaction of light with the tissue. When light is absorbed by tissue at a specific wavelength, it can become excited and scatter. When a biological sample encounters light, it scatters it in different directions. This scattering provides information about the tissue’s structure and composition. Optical imaging analyzes the pattern of scattered light and reconstructs an image of the internal structure [refer to Figure 2]. Many optical imaging techniques rely on fluorescence, where a specific molecule absorbs the light and emits signals in the form of color, graphs, or other visual outputs. Optical imaging methods can also utilize diffraction and diffusion. Diffraction provides high resolution but is limited to superficial layers of tissue, while diffusion-based methods can penetrate deeper but with reduced spatial resolution [24,25].

## 3. Contrast Agents

A contrast agent is a substance that is administered in the human body during medical imaging to enhance the contrast and clarity of specific targets or structures. Depending on the method of imaging being used, it can be taken orally, administered intravenously, or applied to other parts of the body. The basic principle behind these agents lies in their ability to modify how tissues interact with imaging systems, thus improving the ability to differentiate between various types of structures or regions of interest within those areas. Furthermore, they can improve certain imaging techniques like CT scans, MRIs, ultrasounds, and optical imaging in terms of sensitivity, specificity, and resolution [26,27]. For better imaging quality via optical imaging, various types of contrast agents are used such as gold nanoparticles. When gold nanoparticles with anisotropic silica nano shells are used as contrast agents in photoacoustic imaging, high resolution images are produced [28]. Another method utilizes quantum dots, an advanced technology for in vivo imaging, which is particularly valuable for imaging and guidance during surgeries performed on cancer patients. Organic semiconducting agents are used due to their easily tunable properties. This group of agents, which includes small-molecule compounds and nanoparticle derivatives, has been specifically designed for deep-tissue imaging [29]. Additionally, lipid emulsions are used because of their higher reflectivity and improved signal transmission [30].

To improve visibility and contrast of certain targets or tissues in optical imaging, a contrast agent is used, which helps them become more discernible. Contrast agents are chemicals administered intravenously (IV) that interact with the optical imaging system to produce detectable signals. Patients may receive exogenously introduced compounds, such as colored substances or small specks of matter, whereas endogenous symbols sometimes involve naturally present fluorescent or color-bearing substances. Contrast materials allow the differentiation between several components or areas of concern due to their influence on visible tissues’ optical qualities (e.g., to what extent they can absorb/play around with incoming light). In this way, they enhance the sensitivity, specificity, and resolution of methods used for optical imaging (examples include OCT and SPECT), and they can help in the identification of disease and facilitate the observation of cellular and molecular processes [31,32,33,34].

**Figure 3:**
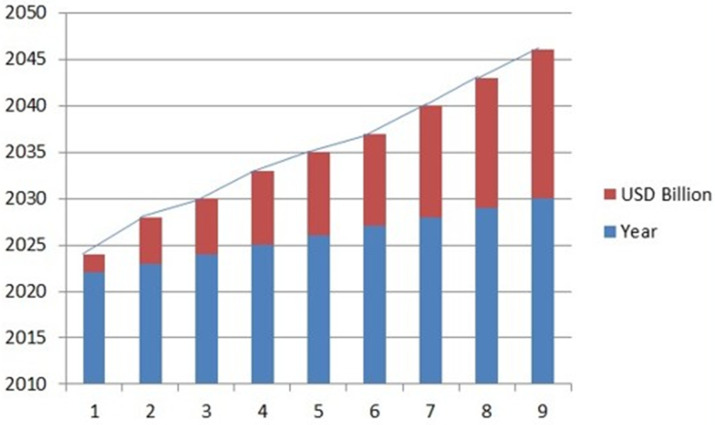
The graph shows CAGR value of the contrast agent from 2022 that depicts that the market value is always increasing (https://www.fnfresearch.com/news/global-mri-contrast-media-agents-market accessed on 3 September 2024) [37].

The market value of the contrast agent is very high, and its global market growth is experiencing significant growth due to the advancements in medical imaging technology and the rising prevalence of chronic disease. The market for contrast agents was estimated to be worth USD 6.28 billion in 2023. It is projected to expand at a 7.98% cumulative annual growth rate (CAGR) to reach around USD 10.74 billion by 2030. Among the primary drivers of this trend are the aging of the global population, the increase in complex comorbidities and chronic illnesses, and the growing prevalence of diagnostic imaging procedures such as CT and MRI scans [refer to Figure 3] [35,36].

## 4. Fluorescence Imaging

Fluorescent imaging is a technique used to visualize sub-cellular components, widely applied in biological sciences and clinical practices. This process involves the use of fluorescent probes or dyes that become excitable when exposed to a light of specific wavelengths. Because of this technology, researchers and clinicians can study cellular metabolism, protein interactions, as well as the course of diseases. For fluorescence imaging, there are different imaging modalities like tomography, endoscopy, or microscopy, and they have the potential to completely change diagnosis and surgery by providing real-time information during surgery and intraoperative decision guidance [38,39].

For fluorescent imaging, we are using certain fluorescent dyes or probes such as fluorescein, which is one of the most common green fluorescent dyes that are brilliant and highly photostable. It is usually labeled with antibodies and nucleic acids. Rhodamine is a red fluorescent dye that is often used to label proteins, nucleic acids, and membranes. The cyanine dyes come in colors such as red (Cy3), far-red (Cy5), and near-infrared (Cy7). They are often used for multiplexing as well as live-cell imaging. Alexa Fluor dyes are fluorescent dyes that have a broad color range and excellent photostability. They are commonly used for immunofluorescence and live-cell imaging. Quantum dots are semiconductor nanoparticles emitting a narrow spectrum and showing strong fluorescence. They are frequently used for multiplexing and prolonged imaging. Indocyanine green (ICG) is a near-infrared fluorochrome used in imaging organs and deep tissues [40,41].

Fluorescence imaging enables the visualization of biological structures and processes with high precision and sensitivity. Fluorescent probes, also known as fluorophores, are used in this process. These probes light up when there is a certain wavelength of light that they respond to. The principles of fluorescence involve excitation and emission. Different wavelengths of light are used to excite various fluorophores, enabling their fluorescence imaging function. Specifically, these fluorophores become activated into higher energy levels via absorption of the excitation light responsible for generation and detection purposes. Therefore, a specific emission wavelength arises from the subsequent release of light at an even longer wavelength than that of the excitation photon. In this way, an image can be obtained through the identification and collection of such emitted light rays. The fluorophores are those types of molecules that can both absorb as well as emit light with varying wavelengths. Such substances are either organic or inorganic, but the organic ones are usually used in biological imaging because they are compatible with live systems. For example, fluorescent proteins or dyes fall under this category of organic fluorophores, while quantum dots serve as inorganic nanoparticles employed for their specific optical properties, and fluorescence is usually visualized by means of light sources that induce excitement in fluorophores. This excitation is followed by using optical filters to distinguish between excitation and emission wavelengths. Finally, after this light collection process, a detector captures the emitted photons. Depending on the specific requirement, a microscope or camera or specialized devices may be used, and fluorescence images use image processing and analysis techniques, which include quantifying fluorescence intensity, determining the localization of multiple fluorophores, removing noise, and subtracting background signals. Advanced approaches such as Fluorescence Lifetime Imaging (FLIM) enable the exploration of molecular interactions at a detailed level [42,43,44,45].

### Parts of Fluorescence Imaging System

The parts of a fluorescence imaging system are described below [46,47,48,49,50,51,52].

Light Source: The light source excites the sample with excitation light and examples of common light sources are lasers, LEDs, and mercury or xenon arc lamps. There are various types of light sources, like xenon arc lamps, which provide broad-spectrum illumination and are commonly used in light microscopy, and the next one is mercury vapor lamps, which are known for emitting intense light at specific wav elengths, which are useful for exciting many standard fluorophores.

Excitation Filter: The excitation filter is placed along the light path to select a specific wavelength that can induce fluorescence in the sample. It only permits the passage of the desired excitation wavelength.

Dichroic Mirror: A dichroic mirror, also known as a beamsplitter, is a device that allows for the passage of fluorescence while reflecting excitation light towards the sample. It separates the light channels leading to excitation and emission.

Objective Lens: The objective lens is responsible for gathering and condensing the emitted fluorescence from the specimen so that it can be viewed on the detector. In addition, it sets the magnification and resolution of the microscope.

Emission Filter: The precise wavelength of fluorescence that fluorophores emit is chosen by inserting an emission filter into the emission light path. As a result, all undesired excitation light is blocked out, and only the desired fluorescence is allowed through.

Detector: The detector gathers the released fluorescence and transforms it into an electrical signal. In general, photomultiplier tubes (PMTs) and charge-coupled devices (CCDs) are the most often used detectors.

Imaging System: The imaging system, consisting of lenses and mirrors, directs fluorescence signals from the sample to the detector, which finally forms an image.

Stage: A stage is a platform that holds and positions a sample under the objective lens, allowing precise user adjustments for focus, illumination intensity, and image acquisition parameters. It also facilitates careful movement and scanning of the specimen.

Control System: The control system includes buttons, knobs, as well as a computer program.

## 5. Types of Fluorescence Imaging

Fluorescence imaging techniques have been utilized in various scientific and medical fields. Several types of fluorescence imaging exist, such as the following.

### 5.1. Widefield Fluorescence Imaging

Widefield fluorescence imaging uses a widefield illumination source that excites the fluorophore present in a sample. A camera or similar device detects emitted fluorescence, therefore giving a two-dimensional view of the sample, and in the collective observations of numerous fluorescent substances; their analytical role in various clinical practices and scholarly investigations is best epitomized by widefield fluorescence imaging. Fluorescence-emitting probes or the natural fluorescence of specific molecules allow the observation of particular cells or tissue structures. Widefield fluorescence imaging is a technique that utilizes the excitation and emission properties of fluorescent molecules. When exposed to certain wavelengths of light known as “excitation light”, fluorescent molecules within the sample absorb the energy and transition into an excited state. After a short period, these excited states return to their original energy levels, releasing excess energy as longer-wavelength fluorescence. This emitted fluorescence is then collected and detected to generate an image.

The advancement of this type of fluorescence imaging has led to high-resolution imaging techniques capable of capturing three-dimensional images with a resolution of up to 100 nm. This was achieved using powerful image reconstruction methods accompanied by unevenly distributed excitation light patterns; optical sectioning that deeply resolved imaging techniques and structured illumination microscopy (SIM) are some of the approaches that have been formulated to create optical sections and enhance the visibility of structures in dense samples [53]. Real-time imaging has high spatiotemporal resolution, and the observation dynamic of biological processes is made possible through the development of wide-field fluorescence imaging technologies for real-time observation [54]. A high-throughput imaging method, such as axial z-sweep capture using 2D projection images, and deep learning-based image restoration, have been proposed to enable high-throughput imaging of complex three-dimensional substances [55]. An improved signal-to-noise ratio and contrast have been achieved using digital micromirror devices in combination with focus sweep attachments. This enhancement results in higher-quality images with better clarity and detail [55].

### 5.2. Confocal Fluorescence Imaging

To improve resolution and optical sectioning, confocal microscopy works by using a pinhole aperture to block out-of-focus light. With this technique, fluorescently tagged samples can be captured in 3D at high resolution. Confocal microscopy is employed in confocal fluorescence imaging, which eliminates out-of-focus light, thus improving image quality. It achieves optical sectioning and improved resolution by a pinhole aperture detecting fluorescence emitted from a specific focal plane after its release into the medium [56]. Throughout the years, confocal fluorescent imaging has evolved greatly. This includes progress in data handling and storage, imaging instruments, fluorescent probes and indicators, and designs of microscopes. Thus, enhanced image quality, increased speed of acquisition, and three-dimensional visualization of changing biological processes have been the outcome of these improvements [56]. And therefore, the use of confocal fluorescence imaging has various advantages. It provides clear three-dimensional images that are less noisy and have more contrast; it also enables optical sectioning, allowing for visualization of specific structures within a sample, and it allows one to analyze the dynamic biological processes in real time. Confocal is different from wide-field imaging as widefield microscopy illuminates the entire sample, leading to faster imaging but sometimes blurry 2D images, whereas confocal microscopy scans a sample point by point and blocks out-of-focus light to provide high-resolution 3D images [57].

### 5.3. Multiphoton Fluorescence Imaging

Multiphoton microscopy enables deeper penetration of tissues and reduces phototoxicity by employing longer-wavelength excitation light, usually in the infrared range. Its common applications include thick sample imaging or live animal imaging [52,58]. High-resolution imaging of biological materials, including living tissues and cells, is made possible by the sophisticated imaging technology known as multiphoton fluorescence microscopy. The technique is predicated on the idea of multiphoton excitation, which is the simultaneous absorption of several photons by a fluorophore that causes the emission of fluorescence. The idea behind multiphoton fluorescence imaging is to stimulate fluorophores using two or more photons rather than simply one. Excitation occurs at the laser beam’s point of convergence, thus allowing for the precise imaging of localized regions within a medium, and the advantages of this technique are deep tissue imaging. This is one of the major benefits of multiphoton fluorescence imaging, which allows for deep penetration into tissues. Multiphoton microscopy can visualize thick specimens due to longer wavelengths of excitation light, which facilitates penetration into tissues and reduces scattering. Nonlinear excitation multiphoton excitation occurs only at the focal point of laser beams, as it is a nonlinear process. Thereby, decreasing background noise and enhancing picture quality are among its inherent optical sectioning abilities; lessened phototoxicity is observed in multiphoton fluorescence imaging as compared to other techniques used for imaging. There is minimal damage to the nearby cells and tissues since the excitation light happens in a relatively tiny area. Quantitative Imaging: Quantitative data regarding cellular and tissue properties can also be obtained by multiphoton fluorescence imaging. This method can be used to quantify several properties, including fluorescence lifespan, intensity, and fluorescence resonance energy transfer (FRET) [59].

### 5.4. Fluorescence Lifetime Imaging Microscopy (FLIM)

This method measures the fluorescence decay time for fluorophores, providing information on the structural changes, environment, and molecular interactions. It is used to study biological functions, protein folding, and protein–protein interactions. FLIM is an effective imaging technique that reveals how long fluorophores last in biological samples. Furthermore, it enables one to see and measure molecular interactions, protein interactions, and biochemical processes in living cells and tissues. The FLIM technique determines the time for which a fluorophore remains in an excited state before emitting a photon. This makes FLIM a reliable and quantitative imaging technique since the fluorescence lifetime does not depend on the excitation or detection efficiency and concentration of the fluorescent molecules used in microscopy. The amount of time a fluorophore stays stimulated before releasing a photon is measured by FLIM [60]. A single photon-sensitive detector, a dichroic mirror, an objective, and a pulsed laser source are necessary parts of a FLIM setup. A frequency-domain lifetime detection module or a time-correlated single-photon counting (TCSPC) system are two examples of modules that FLIM needs in order to measure lifetimes accurately. Fast lifetime picture acquisition is the fundamental benefit of frequency-domain FLIM, which makes it appropriate for dynamic applications like live cell research.

**Figure 4:**
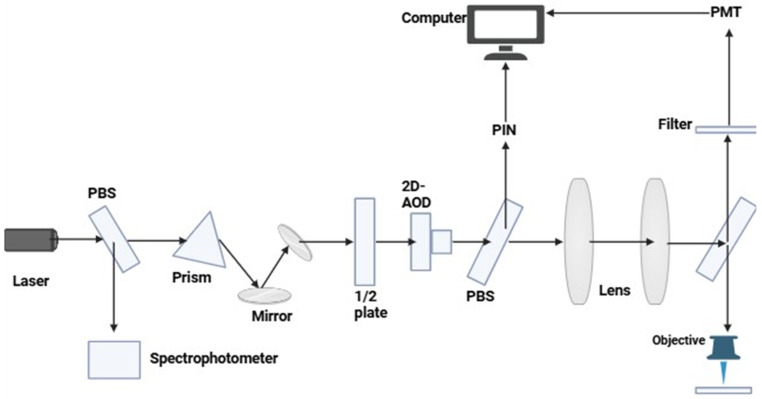
This graphic shows a laser-based optical system, likely a fluorescence lifetime imaging technology, in which the path and polarization of a laser beam are controlled by prism, mirrors, and polarizing beam splitters (PBS). A crucial part, the 2D acousto-optic deflector (AOD), uses sound waves to quickly scan the beam across a sample. Lenses and objective focus the light while a photomultiplier tube (PMT) filters and detects the emitted light from the sample. A computer manages the system and collects data. This entire setup enables precise control all together for detection of light interaction with the sample to facilitate high-resolution imaging [60].

The advantage of this technique is a contrast enhancement that enhances image contrast by providing more information about the fluorophores’ nearby environment. It also uses Förster Resonance Energy Transfer (FRET) to identify protein–protein interactions and to highlight various chemical states. Its application domains are both time-domain and frequency-domain. While frequency-domain FLIM utilizes modulated detectors coupled with sinusoidal modulation of excitation light, time-domain FLIM applies pulse excitation sources together with time-correlated or time-gated detection systems, and quantitative imaging and multiplexing are made possible by FLIM, which enables the multiplexing of fluorescent markers having diverse fluorescence lifetimes but identical emission spectra [refer to Figure 4] [61].

**Figure 5:**
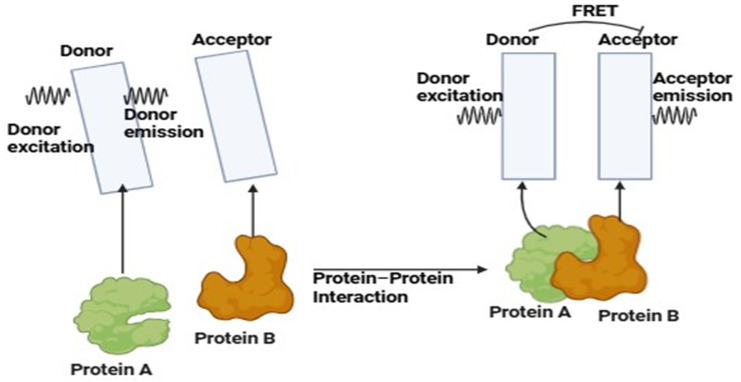
This figure shows Förster Resonance Energy Transfer (FRET), a method for examining molecular interactions, especially those between proteins.

Figure 5 illustrates the FRET—energy transfer mechanism from one neighboring fluorophore to another. It is used to investigate the forms that proteins take on when they bind to other molecules, the ways in which various signals change over time, and the interactions that occur between them [62,63]. Fluorescence resonance energy transfer (FRET) technology has the potential to be a powerful tool for researching the interactions and motions of molecules in biological systems. An excited donor fluorophore gives its energy to a nearby acceptor in a non-radiative manner. FRET is based on the dipole–dipole interactions between acceptor and donor fluorophores. By decreasing donor fluorescence and increasing acceptor fluorescence, the extended dipole mechanism facilitates the energy transfer from one fluorophore to another. The efficiency of fluorescence-based energy transfer is influenced by spectral overlap, distance between the two fluorophores, as well as their orientation [64].

FRET consists of two fluorophores—a donor and an acceptor. It typically emits light at a longer wavelength when the donor fluorophore is stimulated at its particular wavelength. The energy from the excited donor, however, can be non-radioactively transmitted to the acceptor if the acceptor fluorophore is close by (usually within 1–10 nm), giving the acceptor the ability to produce light at its distinctive wavelength. The donor and acceptor are shown apart on the left side of the picture, and the donor is excited to emit light. A protein–protein interaction is shown by the closeness of the donor and acceptor on the right side, which is caused by the interaction of proteins A and B. FRET is the outcome of donor excitation followed by acceptor emission. Therefore, using energy transfer between fluorophores as a basis, FRET acts as a “molecular ruler” to identify interactions [65].

The advantages of this technique are the detection of molecular proximity and interactions, similar to FRET. This technique provides specific information regarding spatial arrangement, conformational changes, and binding events that occur between proteins. FRET enables the investigation of conformational shifts in proteins as well as DNA–protein and protein–protein interactions [64]. This technique is also suitable for measuring nanometer-scale distances using FRET. By measuring energy transfer efficiency, it is possible to determine how far away the donor and acceptor fluorophores are from each other. Therefore, FRET is suitable for studying molecular dynamics and structural changes in living organisms [64]. FRET-based biosensors enable real-time observation of biological processes. These sensors can be directed to specific cell regions and provide real-time data on biochemical processes, such as enzyme activity, ion concentrations, and signaling pathways. The use of FRET-based sensors has significantly advanced the visualization and quantification of molecular events occurring in living cells.

FRET offers an unparalleled degree of sensitivity due to the efficient energy transfer it facilitates between fluorophores. Small changes in molecular interactions can be quantified while analyte concentrations at low levels can still be detected, and a lot of different fluorophores with FRET have been combined together to create multicolor imaging. In this way, multiple molecular events or interactions can be identified at once in one sample [66,67].

### 5.5. Super-Resolution Fluorescence Imaging

Super-resolution fluorescence imaging techniques enable nanoscale imaging by surpassing the diffraction limit of light. Examples of these methods include stimulated emission depletion (STED) microscopy and single molecule localization microscopy (SMLM), both of which provide highly detailed visualization at the molecular level. Therefore, one can observe the small details present in cellular structures more accurately with these approaches. Super-resolution fluorescence imaging is a next-generation microscopy technology that exceeds the optical diffraction barrier of traditional optical microscopy, thus allowing for detailed visualization of structures at the sub-nanometer scale. It has higher spatial resolution and provides insights into cellular activities as well as molecular interactions. Super-resolution fluorescence imaging relies on specialized fluorophores and imaging methods that meet and exceed the diffraction limit. Numerous super-resolution methods have been developed, such as photo-activated localization microscopy (PALM), single-molecule localization microscopy (SMLM), stimulated emission depletion (STED) microscopy, and structured illumination microscopy (SIM). These different methods for achieving super-resolution imaging include patterned illumination, single-molecule localization, or photoactivation, and the advantages are that it has the best possible spatial resolutions. Basically, super-resolution strategies go beyond the diffraction barrier, allowing visualization of features that are just a few nanometers apart. Forensic structural information exposes the arrangement and orientation of different kinds of molecules in cell structures. The use of super-resolution methods in live-cell imaging makes it possible to study dynamic processes in real time. Certain super-resolution approaches allow simultaneous viewing of multiple targets or molecular interactions using various fluorophores [68,69].

### 5.6. In-Vivo Fluorescence Imaging

In vivo fluorescence imaging refers to the observation and monitoring of fluorescently tagged molecules or cells within a living organism. Frequently, it is utilized in preclinical studies that concentrate on gene expression, drug delivery systems, and cancer progress [70,71]. “In vivo fluorescence imaging” is a sophisticated diagnostic method that makes it possible to see and monitor biological processes in living things. It uses fluorescent probes or dyes, which light up when exposed to a particular frequency of light. In vivo fluorescence imaging uses fluorescent labels or probes that cause the emission of fluorescing light to locate them inside cells, tissues, or organ systems and to observe their status and behavior. It is based on the principle of fluorescence, which states that a substance will emit light when excited by a specific wavelength. Important details regarding the fluorescent identifications’ operation and function in bioluminescence are revealed by the spatial information the emitted light provides about their location, distribution, and dynamics.

The advantages of this method include non-invasiveness. In vivo fluorescence imaging enables the visualization of biological processes without the need for invasive procedures such as surgery or tissue sample collection. Its real-time imaging capabilities facilitate observation of dynamic processes such as drug distribution, molecular signaling, and cellular interactions as they occur. It provides valuable new insights into biological processes that were previously unknown. High sensitivity offers in vivo fluorescence imaging to image even where probe concentrations are very low, enabling detection at low levels. This makes it possible to detect small targets or subtle alterations in an organism’s tissues. Specificity: The ability of fluorescent probes to bind to specific targets in a chosen manner enables the imaging of certain cells, tissues, or molecules in a specific way, and multiplexing imaging for multiple targets or molecular interactions within a single organism is achieved through the concurrent use of different fluorescent probes with varying emission wavelengths, and the limitations like phototoxicity, photobleaching create significant challenges when applied to live-cell imaging, and the temporal resolution is also compromised [72].

## 6. Fluorescent Probe Designing and Synthesis

One of the major domains in biological research is the synthesis and production of fluorescent probes. Detecting and visualizing a variety of analytes, such as metal ions, reactive oxygen/nitrogen species, bio thiols, and dangerous gases, can be achieved using fluorescent probes, which are substances emitting light on exposure to light of specific wavelengths [73]. Fluorescent probes are basically designed to include two components: one is a recognition or sensing unit that binds to an analyte of interest selectively, while the other is a fluorescent reporter. The detection unit is responsible for the probe’s sensitivity and selectivity towards the target, whereas the fluorescent reporter part takes care of light emission. Some typical organic fluorescent reporters are naphthalimides, coumarins, and rhodamines. However, there are also other commonly used fluorescent reporters like metal complexes and quantum dots, which are inorganic fluorophores [74,75]. There are different mechanisms that can be used by the recognition unit to interact with the target analyte, such as chelation, hydrogen bonding, and covalent bond formation. Thus, high specificity and sensitivity levels towards the target analyte are dependent on the recognition units [73,74].

Creating effective fluorescent probes involves overcoming several challenges, much like navigating a maze. One major difficulty is achieving a delicate balance between reversibility, kinetics, and selectivity in detection. To prevent cross-reactivity with structurally similar molecules, the probe must selectively interact with the target analyte under physiological conditions. Additionally, because analyte concentrations—such as H_2_O_2_—are typically low, the detection reaction should exhibit fast kinetics to enable real-time or near-real-time signal detection. While irreversible reactions are common, reversible reactions are preferable for longitudinal tracking and quantitative analysis. The design process becomes even more complex when selecting fluorophores with large Stokes shifts, high quantum yields, and optimal wavelengths. Further challenges arise from cellular barriers, including solubility issues, membrane permeability, and potential protein interference. Lastly, ensuring organelle specificity, minimizing probe turnover, and optimizing clearance are crucial for obtaining accurate and reliable results. To fully harness the potential of fluorescence probes in biological research and medical diagnostics, researchers must navigate these obstacles effectively [76].

The creation of a fluorescent probe intended for a specific analyte must consider the following factors: Selectivity means the probe must have minimal cross-reactivity with other species in the sample and high selectivity for the target analyte. A recognition unit that can attach to the target analyte is often incorporated, using methods such as chelation, hydrogen bonding, or covalent bond formation as a means of achieving this. Sensitivity means that for probe detection, targeted analytes must have a lower concentration. This depends on the brightness, which is a function of the fluorescent reporter groups’ molar extinction coefficient and quantum yield. The reason is that brighter probes allow for deeper penetration, hence decreasing detection limits [73]. Photostability means that for long-term imaging and quantification, a photobleaching-resistant probe must be used. The design of the probe and choice of fluorescent reporter group are determinants of photostability [73]. Permeability of cells and solubility is also an important factor for biological uses; the investigation must be water-soluble and can penetrate cell membranes. The solubility and cell permeability of the probe can be tuned by including suitably chosen functional groups and linkers. Compatibility with biological systems means the non-toxic nature of the probes is required at doses employed for obtaining images from them in cells or tissues [74]. The fluorescent reporter group choice, as well as the design of a probe, may potentially determine biocompatibility, and self-assembly simplicity should be easy, and one ought to take only a few simple steps for obtaining the probe from commercially available beginning ingredients. In this way, faster construction and improvement of recent probes become achievable. A fluorescent reporter that releases light when it binds to a particular biomarker is combined with a recognition unit that binds to the biomarker selectively to create fluorescent probes. High specificity and sensitivity are guaranteed by this architecture, enabling accurate identification and visualization of disease-related compounds in biological samples. Probes that employ chelation, hydrogen bonding, or covalent bond formation have very little cross-reactivity with other species.

Fluorescent probe manufacturing typically involves a few processes, such as preparing the fluorescent reporter group, the recognition unit, and the final probe molecule. For example, a naphthalimide-based fluorescent probe is made by following these procedures to detect Cu^2+^ ions [73,77]: the synthesis of 6-bromo-2-butyl-benzo [de]isoquinoline-1, 3-dione from N-butylamine and 4-bromo-1,8 naphthalic anhydride yields (A). Compound A reacts with hydrazine hydrate to produce 6-hydrazino-benzo [de]isoquinoline-1,3-diones (B). Complement B was coupled to a suitable recognition entity, such as the thiophene moiety, to produce the final fluorescent probe L. The selectivity of the above-synthesized probe L towards Cu^2+^ ions was examined using a variety of spectroscopic methods, including fluorescence spectroscopy and UV–visible absorption. Observing intracellular cupric ions in living cells is another use for it.

A new fluorescent probe called NA-LCX was logically created and synthesized in a recent work to detect hydrogen sulfide (H_2_S) in biological systems. This study addressed the requirement for sensitive and selective measuring techniques because H_2_S plays a crucial function as a signaling molecule. This design’s foundation was a framework of diformylphenol and hydroxyl-naphthalene, which allowed for coordination with Cu^2+^ ions and improved H_2_S detection’s sensitivity and specificity [78]. NMR and mass spectrometry confirmed that the NA-LCX ligand was produced by dissolving 3-hydroxy-2-naphthoyl hydrazide and 2,6-diformyl-4-methylphenol in ethanol and then refluxing the mixture for six hours. The NA-LCX-Cu^2+^ complex was created when the ligand reacted with Cu(ClO_4_)_2_·6H_2_O in methanol. When H_2_S was added, the complex showed a significant “on-off-on” fluorescence response, suggesting that the probe had successfully released Cu^2+^ ions and returned to its initial fluorescence. The efficacy of the probe was demonstrated by the determination of the detection limit for H_2_S at 2.79 μM. Furthermore, NA-LCX’s promise for in vivo applications without significant cytotoxicity was confirmed by cell imaging investigations that showed it could detect both Cu^2+^ and H_2_S in human liver cancer cells (HepG-2). Overall, the study shows how to successfully apply rational design principles to create a fluorescent probe that greatly advances research in cellular biology and biochemistry, especially in figuring out how H_2_S functions in physiological processes [78,79].

## 7. Applications of Fluorescence Probes

### 7.1. Molecular Imaging and Cancer Detection

Molecular imaging relies heavily on fluorescence imaging; it is a key instrument in this context since it enables one to view without invasive methods what occurs inside animals’ bodies at the cellular level, including even very small quantities of drugs. Thus, some notable aspects of molecular imaging using fluorescence are outlined below [refer to Table 1].

Fluorescent molecule imaging has been used extensively in biological medical applications as a non-invasive method of examining diseases, researching biological mechanisms, and studying drug actions. It is particularly useful for cancer detection probes, which have high specificity and sensitivity for detecting cancer in preclinical animals and aid in oncological surgeries. Improved tumor boundary identification can be achieved through advanced methods like structured illumination fluorescence imaging. The development of probes is crucial for specific imaging applications. Such probes that emit far-red to near-infrared light are of great value because they can penetrate tissues deeply and have a high sensitivity. These probes have impressive fluorescence enhancements and low detection limits; thus, they immediately respond to specific targets, like HOCl [85]. Clinicians now have access to real-time imaging through fluorescence imaging, which is increasingly being adopted in clinical settings. Advanced adaptive methods and visualization techniques are making fluorescence imaging more precise and robust in the clinics [86], Surgical guidance that provides real-time feedback to surgeons and intraoperative fluorescence molecular imaging improves endoscopic and surgical imaging. Fast imaging techniques such as optical flow correction enhance sensitivity while reducing motion artifacts [82]. Near-infrared imaging is highly valuable for cancer detection due to its excellent spatial resolution, real-time visualization, and ability to profile multiple molecules; since optical imaging provides non-invasive ways to observe and track cellular activity, it is becoming more and more important in the diagnosis and treatment of cancer. By evaluating the molecular etiology and increasing tumor border delineation, it helps diagnose diseases accurately and increases the sensitivity and specificity of cancer detection. Because they may penetrate deep tissue, methods like near-infrared imaging are essential for screening for malignancies like breast cancer. By using fluorescence molecular imaging, optical imaging also facilitates postoperative monitoring, intraoperative guiding, and preoperative planning. It helps with accurate surgical resection by tracking gene expression, disease progression, and therapy response. Optical imaging can transform cancer treatment and enhance patient outcomes by integrating structural, functional, and molecular data [87]. It overcomes the limitations of conventional techniques used for tumor identification, lymphatic imaging, in vivo cancer imaging, and surgical guidance. Fluorescence imaging methods, utilizing tumor-avid probes with high sensitivity and specificity for malignancy detection, are widely employed in preclinical models and oncological procedures. Fluorescence imaging techniques have made significant progress in the field of molecular imaging in the context of cancer detection and treatment. Strategies such as structured illumination fluorescence imaging, which enhances tumor boundary delineation and thereby improves tumor identification accuracy, are also mentioned. During surgery, a novel near-infrared (NIR) fluorescence probe designed for real-time tumor imaging was used in one well-known case study. With a technique that involves strong fluorescence upon attachment to cells expressing tumor markers, like folate receptors, this conjugated polymer-based probe demonstrated very selective targeting of cancer cells. According to the study, fluorescence imaging improves the accuracy of tumor removal by giving surgeons real-time feedback during surgical resections, allowing for better differentiation between cancerous and healthy tissue. This imaging modality’s application in clinical trials involving patients with ovarian cancer resulted in a significant decrease in positive surgical margins; reported figures show a decrease from roughly 20% to less than 5%, highlighting its potential to improve surgical outcomes and lower cancer recurrence rates. Additionally, these studies showed that combination therapies using NIR probes and the name of the manufacturer are Pike technologies located in Madison, Wisconsin, United State, tecUSA located in New York, United States etc and fluorescence-guided surgery not only improved visualization but also made it easier to identify micro-metastases that would otherwise go undetected, highlighting the crucial role fluorescence imaging will play in onco-surgery and customized cancer treatment plans in the future [81,84].

### 7.2. Brain and Cardiovascular Imaging

Fluorescence imaging plays a critical role in cardiovascular and vascular imaging, along with enabling one to visualize the structures and workings of the brain. Some key points on how fluorescence imaging is being utilized in different fields include the following:

Brain Imaging: Fluorescence imaging techniques allow researchers to investigate cellular and molecular processes within the central nervous system (CNS). It is possible for them to track dynamic events occurring in the brain via custom-made optics or fluorescent tags. Neurotransmission, synaptic communication, as well as molecular dynamics can be visualized using this imaging method, providing valuable information on brain functioning and neurological diseases [82]. Fluorescence imaging provides a means of visualizing the cerebral vasculature without requiring invasive surgeries such as craniotomies. By utilizing specific agents, innate near-infrared photoluminescence, fluorescence imaging allows fine monitoring of blood vessels by penetrating deep into brain tissues. This non-invasive method makes it possible to track and analyze vascular changes and adjustments in real time, which is crucial for comprehending illnesses like strokes. Additionally, it makes it possible to evaluate irregularities in blood flow and blood perfusion [83,88], and near-infrared fluorescence imaging, particularly in the second near-infrared spectral band (NIR-II), provides great spatial resolution and deep tissue penetration for cardiovascular imaging. Because it uses FDA-approved materials like indocyanine green (ICG), this imaging technique is perfect for use in clinical cardiovascular imaging [89]. Cerebrovascular disease could be diagnosed and treated using macro-imaging and micro-angiography. Deep monitoring of the cerebral vasculature is made possible by NIR-II fluorescence imaging, which enables the detection of any abnormalities or modifications in vascular structure [90,91].

Both brain and cardiovascular studies have benefited greatly from fluorescence imaging, which has improved our comprehension of biological processes and disease mechanisms. In one prominent example study, neuronal activity was tracked in vivo using genetically encoded calcium indicators (GECIs) in brain imaging. By using sophisticated two-photon imaging techniques, scientists were able to see calcium dynamics in real time throughout several different parts of the living mouse brain. Neural activity during sensory stimulation and behavioral activities could be tracked thanks to the use of the GCaMP family of indicators, where calcium ion binding produced a detectable increase in fluorescence. This method has provided novel insights into the complex dynamics of learning and memory by revealing how brain circuits process information [91].

Similarly, a groundbreaking study in cardiovascular imaging created a fluorescent cardiac imaging system that uses indocyanine green (ICG) to measure blood flow in real time during surgery. High-resolution intraoperative imaging was made possible by this innovative technique which allowed surgeons to accurately observe and measure coronary artery blood flow. To maximize the signal from ICG, the implementation required carefully planned optical setups furnished with LED light sources and appropriate filters. The system’s remarkable correlation values of 0.9938 for blood flow data in preclinical studies confirmed its effectiveness in detecting myocardial perfusion problems during coronary artery bypass graft procedures. This technology significantly enhances the surgical decision-making process, ultimately leading to improved patient outcomes [92].

Table 2 describes the benefits and difficulties of fluorescent probe-based optical imaging [refer to Table 2].

Moreover, fluorescent probe optical imaging faces challenges such as tissue autofluorescence, which can obscure the signal from the probes, and limited depth penetration due to light scattering and absorption by biological tissues. These issues complicate obtaining clear and accurate images from deeper tissue layers. Tissue autofluorescence interferes with signal detection in fluorescence imaging, lowering the signal-to-background ratio. A purified diet, longer excitation/emission wavelengths, and NIR-II imaging are among the methods that can be employed to solve this issue [73].

Autofluorescence, an inherent feature of biological tissues, results from endogenous fluorophores and greatly hinders optical imaging by producing background noise. The kind of tissue and the wavelengths of excitation and emission affect these phenomena, which is exacerbated by substances like riboflavin and NADH [95]. Strategic methods can lessen these impacts, though. Spectral overlap is reduced by using dyes that emit far-red or near-infrared light. Controlling one’s diet, particularly by consuming less chlorophyll, reduces autofluorescence in the digestive system. Cutting-edge methods like spectral unfixing and time-resolved imaging facilitate signal separation. Increasing the image window to NIR-II (1000–1700 nm) further minimizes autofluorescence and scattering. In order to effectively handle autofluorescence and optimize biomedical optical imaging, a multimodal approach that includes spectrum changes, dietary alterations, and sophisticated imaging is used [96].

The restricted depth of tissue penetration, the variable characteristics of fluorescent dyes, and the requirement for standardized imaging methods are some of the challenges facing the adoption of fluorescence-based optical imaging in clinical settings. The development of quantitative techniques and interdisciplinary cooperation are necessary to address these issues and guarantee accurate and repeatable results for regulatory approval. Because of these efforts, optical fluorescence imaging will eventually become a basic part of standard care [97].

Depth Penetration: The significant light scattering and autofluorescence that occur between 650 and 900 nm limit the ability to image deep tissues using conventional optical techniques. With documented depths through tissue at roughly 3.2 cm, NIR-II imaging (1000–1700 nm) offers superior depth penetration in response to such limitations [98,99].

### 7.3. Clinical Translation

Optical imaging in biomedical applications has become highly effective with fluorescent probes, enabling real-time viewing of cellular activities and ultimately facilitating improved disease detection and therapy, particularly for cancers. Key considerations for clinical translation are designing fluorescent probes; it is important to consider clinical translation. To achieve success in in vivo performance, several key factors must be considered, including stability specificity, clearance characteristics, and effective urine excretion. Compared to NIR-II (1000–1700 nm) probes, which exhibit superior intravital performance due to lower tissue autofluorescence, better signal-to-noise ratios, and deeper penetration depths, those emitting light in the visible and near-infrared (NIR)-I range (700–900 nm) have undergone clinical trials and there are several biomedical applications for fluorescent probes, such as drug delivery, biological imaging, medical diagnosis, and therapy, among others. High performance in vivo imaging has been demonstrated using NIR-II organic small molecule probes with emission maxima exceeding 1200 nm [96,100].

Despite their potential, only a few fluorescent probes have been approved for medical imaging. This presents challenges, including limited clinical acceptability. The delay is primarily due to the complexity of probe development, regulatory hurdles, and the need for extensive validation in clinical settings [101]. FTS (fluorescence-traced surgery) [21] is one of the primary applications of fluorescent probes. By enabling real-time tumor visualization, this technique allows surgeons to achieve more precise tumor removal while minimizing damage to surrounding healthy tissues [21]. Fluorescent probes must undergo rigorous preclinical and clinical testing before receiving regulatory approval.

An important advancement in the therapeutic use of fluorescence imaging is the use of indocyanine green (ICG) in fluorescence-guided surgery (FGS) for various cancer types [102]. In a groundbreaking clinical investigation, researchers investigated the potential of intraoperative near-infrared fluorescence imaging to enhance visibility during lymph node dissection in patients with breast cancer and melanoma. The study found that when compared to traditional approaches, ICG administration significantly increased detection rates by enabling the real-time identification of sentinel lymph nodes. Specifically, ICG fluorescence aided in determining the exact position of lymphatic channels, allowing for successful sentinel lymph node mapping in 95% of participants, which is a significant improvement over the usual 75% attained with conventional methods. The clinical results also showed that the incorporation of fluorescence imaging reduced surgical problems and enhanced oncological results in addition to improving the surgical procedure’s technical success. This case study demonstrates how fluorescence imaging technologies, especially ICG, can revolutionize surgical practice by increasing the precision of cancer detection and treatment, which will encourage a wider use of fluorescence-based methods in clinical oncology [103,104].

## 8. Conclusions

In conclusion, optical imaging, especially when combined with fluorescent probes, represents a revolutionary advancement in therapeutics and medical diagnostics. This non-invasive imaging technique allows visualization of biological structures and diagnosis of cellular and molecular activities within the human body using specific characteristics of light. In addition, high resolution of this imaging method permits real-time monitoring of cancer and other diseases efficiently without using adverse ionizing radiation. Thes imaging capabilities are further improved by the advancements in optical contrast agents, particularly fluorescent probes, which make it possible to target biomarkers selectively and to significantly improve imaging sensitivity and specificity. This development could transform clinical diagnosis and treatments, especially in the fields of neurology and oncology, and opens the door for the early diagnosis of anomalies. Despite numerous advancements, there are several limitations in these technologies’ clinical translation. Only a small number of fluorescent probes have made the leap to routine clinical use, although several studies have demonstrated encouraging outcomes in preclinical trials due to difficulties including the need for systematic validation to guarantee safety and efficacy, intricacy of creating efficient probes, and strict regulatory constraints. Moreover, issues such as tissue autofluorescence and limited depth penetration of optical imaging techniques significantly hinder the broader application of these technologies.

However, these gaps could be addressed in the future by designing and developing highly biocompatible novel fluorescent probes with better signal-to-noise ratios particularly in the NIR-II region to enhance selectivity and deeper tissue penetration. Furthermore, incorporation of cutting-edge imaging technology, such as machine learning algorithms, could improve image processing and substantially yield priceless insights of intricate biological processes. Additionally, it is very essential that regulatory agencies, physicians, and scientists work together to expedite the approval procedures for novel imaging agents. In addition, the production of highly efficient fluorescent probes that can address complex issues is very crucial to improving imaging techniques’ sensitivity and specificity and hastening the field’s transition to clinical applications. Ultimately, through coordinated efforts and inclusive multidisciplinary research, optical imaging could become a fundamental component of modern medical diagnostics and treatment, leading to better patient outcomes.

## Figures and Tables

**Table 1:** An overview of the uses of fluorescence molecular imaging in medical research and diagnostics.

Application	Description
Fluorescence molecular imaging	A non-invasive technique for tracking illnesses, researching biological processes, and learning about how drugs work [80].
Cancer identification	Improves tumor border delineation with sophisticated imaging techniques; uses tumor-avid probes for high specificity and sensitivity in detecting malignancies [81].
Development of probes	The development of fluorescent probes that glow in the far-red to near-infrared spectrum and are sensitive to specific targets like HOCl has allowed for deep tissue penetration and high sensitivity.
Real-time imaging	With the use of visualization techniques and adaptive procedures to improve accuracy, fluorescence imaging is increasingly being used in clinical settings.
Surgical guidance	Enhance endoscopic and surgical imaging by continuously providing feedback during the procedure. Motion artifacts are minimized by using methods such optical flow correction.
Near–infrared imaging	Provides superior real-time display and spatial resolution for cancer diagnosis, making up for the drawbacks of conventional imaging modalities in a range of applications.
Brain imaging	Enables the cellular and molecular analysis of brain activity, using specialized optics and fluorescent markers to examine neurotransmission and synaptic communication [82].
Vascular imaging	Non-invasive cerebral vasculature observation is vital to comprehending disorders such as stroke since it tracks anomalies in blood vessels in real time [83].
Cardiovascular imaging	To assess vascular anatomy and detect cerebrovascular diseases, employ near-infrared fluorescence imaging, which offers deep tissue penetration and great spatial resolution [84].

**Table 2:** Benefits and difficulties of fluorescent probe optical imaging.

Benefits	Difficulties
Real-time imaging: Fluorescent probes enable the visualization physiological conditions and real-time cellular operations [93].	Background signals: Fluorescence signals resulting from natural cofactors within living cells might provide a problem for imaging research [94].
High sensitivity: The detection of specific biomolecules is made possible by the high sensitivity and specificity of fluorescent probes [94].	Elevated background signals: They can diminish signal contrast in intact tissue and multi-cell systems [94].
Non-invasive imaging: Highly precise non-invasive imaging of cellular events is made possible by tiny fluorophores [93].	Challenges with in vivo cancer imaging: Creating fluorescent nanoparticle probes presents difficulties.
Increased functionality: Optimal optical characteristics for certain subcellular locations are provided by small-molecule fluorescent probes.	Complex photo physical schemes: The complex photo physical schemes of certain fluorescent probes influence their bio-analytical responses.
Targeted therapy: Fluorescent probes can help medications be delivered in a specific manner in targeted therapy.	Inadequate signal-to-background ratios hinder the clinical application of optical molecular imaging.

## Data Availability

No new data were created or analyzed in this study. Data sharing is not applicable to this article.
